# Crystal Chemistry of a Nickelate Superconductor: La/Pr Distribution, Oxygen Stoichiometry, and High-Pressure Structural Evolution in La_2_PrNi_2_O_7_

**DOI:** 10.1021/acs.inorgchem.6c03000

**Published:** 2026-10-08

**Authors:** Javier Gainza, Lifen Shi, Gang Wang, Ningning Wang, María Teresa Fernández-Díaz, Stanislav Savvin, Jinguang Cheng, José Antonio Alonso

**Affiliations:** † 55553 Instituto de Ciencia de Materiales de Madrid (ICMM-CSIC), c/Sor Juana Inés de la Cruz 3, Cantoblanco, Madrid 28049, Spain; ‡ European Synchrotron Radiation Facility (ESRF), 71 Avenue des Martyrs, CS40220, Grenoble 38043, France; § Beijing National Laboratory for Condensed Matter Physics, 47849 Institute of Physics, Chinese Academy of Sciences, Beijing 100190, China; ∥ Max Planck Institute for Chemical Physics of Solids, Dresden 01187, Germany; ⊥ 56053 Institut Laue-Langevin (ILL), 71 Avenue des Martyrs, BP 156, Grenoble Cedex 9 38042, France

## Abstract

We report a comprehensive structural characterization of La_2_PrNi_2_O_7_, a bilayer Ruddlesden–Popper nickelate that has been described to exhibit high-temperature superconductivity under hydrostatic pressure. Synchrotron X-ray diffraction (SXRD) data and high-resolution neutron powder diffraction (NPD) data, collected at ambient pressure, together with NPD data recorded under pressures up to 8.42 GPa in a Paris–Edinburgh cell, have been analyzed by the Rietveld method in the orthorhombic *Amam* space group (No. 63). This work aims at providing precise bulk structural benchmarks—site-selective cation distribution, oxygen stoichiometry, and the pressure evolution of the Ni–O–Ni bond geometry—that are essential inputs for theoretical models. High-resolution NPD, which offers a strong La/Pr scattering-length contrast, yields refined La/Pr occupancies that suggest a slight preference of Pr for the outer rock-salt site (La2/Pr2), although the deviation from a random distribution does not exceed three standard deviations and is therefore not statistically conclusive. NPD also assesses the full oxygen stoichiometry of the specimen under study. Under applied pressure up to 8.42 GPa (below the superconducting critical pressure *P*_c_ ≈ 11 GPa), the orthorhombic structure is retained with a systematic compression of all lattice parameters, a reduction of the unit-cell volume of ∼4.9%, and a progressive straightening of the out-of-plane Ni–O–Ni bond angle. No structural phase transition to the tetragonal *I4*/*mmm* symmetry is observed in the pressure range studied, consistent with the superconducting onset occurring above *P*_c_ ≈ 11 GPa. Cooling to 5 K at 8.42 GPa produces no measurable additional structural change, indicating that precursor distortions to the superconducting state are subtle.

## Graphical abstract

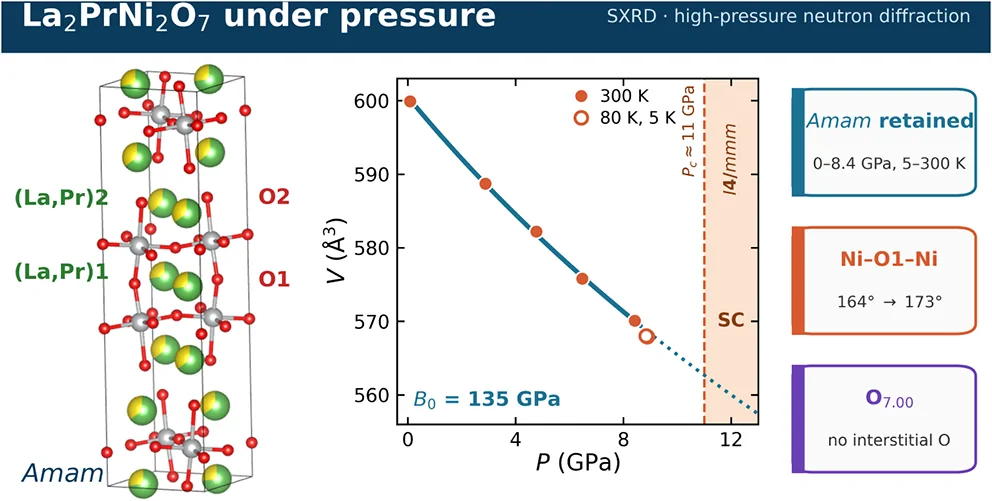

## 1 Introduction

The discovery of high-temperature superconductivity in pressurized La_3_Ni_2_O_7_ by Sun in 2023, with an onset temperature *T*_c_ ≈ 80 K, has generated extraordinary interest because it represents the second class of unconventional high-*T*_c_ superconductors in the liquid-nitrogen temperature range.^1,2^ La_3_Ni_2_O_7_ belongs to the Ruddlesden–Popper (RP) series La_*n*+1_Ni_*n*_O_3*n*+1_ (*n* = 2), where corner-sharing NiO_6_ octahedra are assembled in bilayer blocks separated by interleaved rock-salt LaO layers.^3,4^ At ambient conditions, the compound adopts an orthorhombic *Amam* (No. 63) structure arising from cooperative tilting of the NiO_6_ octahedra, and under high pressure (HP), the structure transforms to tetragonal *I4*/*mmm* symmetry, concomitant with the emergence of superconductivity.^1,5,6^

The sensitivity of the superconducting (SC) phase to subtle changes in the crystal chemistry has been clearly established. Isovalent Pr substitution in La_3–*x*_Pr_*x*_Ni_2_O_7_ dramatically enhances the phase purity and bulk SC volume fraction.^7,8^ Wang demonstrated that superconductivity appears in La_2_PrNi_2_O_7_ (*x* = 1) above *P*_c_ ∼ 11 GPa and achieves ∼97% SC volume fraction at *P* ≈ 18 GPa, with *T*_c_ ≈78–82 K.^7^ The remarkable improvement has been attributed primarily to the suppression of the intergrowth with other R–P phases and the near-stoichiometric oxygen content facilitated by Pr doping.^9,10^ Smaller Pr^3+^ cations preferentially occupy the outer rock-salt La sites, expanding the interstitial volume and reducing inner apical oxygen (O1) vacancies that are known to suppress superconductivity.^9,11,12^

Oxygen stoichiometry is thus a crucial control parameter. High-pressure oxygen annealing (HPO, >200 bar) at 500–600 °C introduces interstitial oxygen atoms that form ordered stripe-like superstructures with space group *B2em* (No. 39), suppressing superconductivity even at 14 GPa.^9^ This behavior contrasts strikingly with the cuprate analogues, where ordered interstitial oxygen in La_2_CuO_4+δ_ promotes superconductivity by hole doping the d_*x*2*–y*2_ band.^13,14^ The dichotomy reflects the orbital selectivity of nickelates, where self-doping of both d*_z_*_2_ and d_*x*2*–y*2_ orbitals gives rise to a more complex landscape of competing orders.^15–18^

High-pressure structural studies on La_3–*x*_R_*x*_Ni_2_O_7_ have been performed predominantly by synchrotron X-ray diffraction (SXRD),^5,6,19^ which cannot precisely locate light oxygen atoms or resolve small differences in Ni–O bond lengths and angles. Neutron powder diffraction (NPD) under pressure provides the complementary structural information indispensable for theoretical modeling of the superconducting pairing mechanism.^20,21^ Neutron powder diffraction combined with muon-spin rotation has also been used to determine the spin-density-wave order that develops in La_3_Ni_2_O_7_ and La_2_PrNi_2_O_7_ at ambient pressure below about 150 K.^22^ The out-of-plane Ni–O–Ni bond angle, which evolves toward 180° in the high-symmetry tetragonal phase, is a key parameter controlling the bandwidth of the flat d*_z_*_2_ bonding band at the Fermi level.^23,24^ Despite the importance of these data, a detailed HP-NPD study of La_2_PrNi_2_O_7_ is still lacking.

In this work, we present a multi-technique structural study of La_2_PrNi_2_O_7_ combining (i) high-resolution SXRD at ambient pressure to determine the Pr/La site distribution and anisotropic displacement parameters, (ii) high-resolution NPD at ambient pressure to assess oxygen stoichiometry with high sensitivity, and (iii) HP-NPD up to 8.42 GPa and down to 5 K to track the pressure and temperature evolution of the structure toward the superconducting phase. All data were analyzed by the Rietveld method implemented in FullProf.2k.^25^

## 2 Experimental Section

### 2.1 Sample Preparation

Polycrystalline La_2_PrNi_2_O_7_ was synthesized by the sol–gel (Pechini) method.^7,8^ Stoichiometric amounts of La_2_O_3_ (99.99%, Alfa Aesar), Pr_6_O_11_ (99.9%, Alfa Aesar), and Ni­(NO_3_)_2_·6H_2_O (99.99%, Alfa Aesar) were dissolved in deionized water with citric acid (≥99%, Sigma-Aldrich) and nitric acid (65%, Sigma-Aldrich), which were added in appropriate proportions. The resulting solution was stirred in a 90 °C water bath for approximately 4 h to form a homogeneous green nitrate gel. The gel was calcined overnight at 800 °C to remove excess organic matter, then ground, pressed into pellets, and sintered in air at 1100–1150 °C for 24 h. Phase purity was confirmed by laboratory X-ray powder diffraction in a Bruker D8 Advance diffractometer (Cu Kα radiation, Bragg–Brentano geometry) before transferring the sample to neutron and synchrotron facilities. The sample used in this study was prepared following the above-described Pechini protocol but represents an independently synthesized batch; its structural identity with the superconducting material was confirmed by comparison of laboratory XRD patterns and lattice parameters.

### 2.2 Synchrotron X-ray Diffraction

High-resolution SXRD data were collected at the ID22 beamline of the European Synchrotron Radiation Facility (ESRF, Grenoble) at room temperature. The wavelength was λ = 0.40004 Å. The instrument operated in a parallel-beam geometry with a multianalyzer stage between the sample and a Dectris Eiger2 X 2M-W CdTe pixel detector, providing extremely high angular resolution (Δ*Q*/*Q* ≈ 10^–4^). The angular range was 0.007–62.04° (2θ), with a step size of 0.001°. The sample (∼10 mg) was loaded in a 0.5 mm glass capillary which was spinning during the acquisition. A total of 2977 independent reflections were included in the Rietveld refinement.

### 2.3 Neutron Powder Diffraction at Ambient Pressure

NPD data at ambient pressure were collected on the D2B high-resolution powder diffractometer at the ILL, Grenoble (λ = 1.594 Å, take-off angle = 135°). The angular range was 0.05–159.95° (2θ), with a step size of 0.05°. Approximately 1 g of sample was loaded in a vanadium container. Due to the high coherent scattering length of oxygen (*b*(O) = 5.803 fm), NPD is significantly more sensitive to the oxygen sublattice than SXRD, enabling the refinement of occupancy factors of oxygen atoms. A total of 347 independent reflections were used in the Rietveld refinement at ambient pressure. The angular region of 158.5–160.5° in 2θ, which contains a weak reflection from the vanadium sample container, was excluded from the refinement.

### 2.4 High-Pressure Neutron Powder Diffraction

HP-NPD experiments were performed at the D20 high-flux powder diffractometer at the ILL (λ = 1.543 Å, take-off angle = 90°). A Paris–Edinburgh (PE) press equipped with cubic boron nitride (cBN) anvils was used to generate pressures up to ∼8.5 GPa. The sample (∼100 mg) was loaded in a zero-scattering Ti–Zr alloy gasket with a 4:1 methanol–ethanol mixture as the hydrostatic pressure-transmitting medium. 5% NaCl (sodium chloride) powder was coloaded as an in situ pressure gauge; the actual pressure at each point was calibrated from the Rietveld-refined lattice parameter of the NaCl *Fm3̅m* phase using the established equation of state.^26,27^

Data at D20 were collected at room temperature (RT) at nominal PE press loadings of 120, 600, 880, 1200, and 1500 bar, corresponding to calibrated pressures of 0.08, 2.88, 4.77, 6.47, and 8.42 GPa, respectively. After the room-temperature pressure series, the sample was cooled to 80 K (1500 bar) and then to 5 K (1500 bar), corresponding to 8.88 and 8.84 GPa, respectively. Each pattern required 3–8 h counting time; longer counting times were needed at high pressure due to the reduction in irradiated sample volume as the anvil gap decreases. The pressure (bar) values quoted refer to the nominal hydraulic load applied to the PE press ram; the actual sample pressures are determined by the NaCl gauge and take into account unisostaticity and friction losses.

### 2.5 Structure Refinements

All data were analyzed by the Rietveld method as implemented in FullProf.2k (Version 7.95, January 2023).^25^ The Thompson–Cox–Hastings pseudo-Voigt profile function was used for all data sets. For the HP-NPD data, the orthorhombic *Cmcm* space group (No. 63, standard setting) was used in the Rietveld refinement; this is equivalent to *Amam* by a cyclic permutation of axes (*aCm* → *cAm*, *bCm* → *aAm*, *cCm* → *bAm*). All structural parameters reported in this paper are expressed in the nonstandard *Amam* setting, selected because the long *c*-axis is vertical. Isotropic displacement parameters (*B*_iso_) were refined for all atoms. La/Pr mixed occupancies were refined with the constraint that the total occupancy sums to the nominal stoichiometry; for the ambient-pressure SXRD refinement, the four fractional site occupancies (La1, Pr1, La2, Pr2) were treated as one independent variable reflecting the overall La:Pr = 2:1 ratio. The SXRD pattern was refined as a two-phase model: a minor La_2_NiO_4_ impurity (space group *Fmmm*, No. 69) was identified from the weak reflections at 6.2° and 8.1° 2θ, indexed as 111 and 113, and was included in all subsequent refinements; its refined weight fraction is 0.49(3)%. In addition, an anisotropic strain-broadening model based on the direction-cosine formalism implemented in FullProf was applied to the main phase in order to describe the reflection-dependent broadening arising from the layered morphology of the Ruddlesden–Popper structure. Occupancies are reported throughout as site-normalized fractions, i.e., unity for a fully occupied site, with the two La/Pr fractions adding up to 1.000 at each mixed site. The NaCl phase was included in all HP-NPD refinements as a secondary phase.

## 3 Results and Discussion

### 3.1 Crystal Structure at Ambient Pressure from SXRD

La_2_PrNi_2_O_7_ crystallizes at ambient conditions in the orthorhombic *Amam* space group (No. 63, *Z* = 4), with unit-cell parameters *a* = 5.3810(3) Å, *b* = 5.4543(4) Å, *c* = 20.417(1) Å, *V* = 599.22(7) Å^3^ (Table 1a, SXRD). These values are in excellent agreement with those reported by Wang (*a* = 5.389(1) Å, *b* = 5.444(1) Å, *c* = 20.425(5) Å)^7^ and with own results from D2B neutron diffraction (*a* = 5.3769(1) Å, *b* = 5.4548(2) Å, *c* = 20.3990(8) Å, Table 1b, NPD). The slight differences between SXRD and NPD values reflect the complementary sensitivity of the two probes to lattice parameters in the presence of a small orthorhombic distortion.

**Table 1:** Structural Parameters of La_2_PrNi_2_O_7_ at Ambient Pressure and 300 K, Space Group *Amam* (No. 63), *Z* = 4, Refined from (a) Synchrotron X-ray Diffraction Data (ID22, ESRF) and (b) Neutron Powder Diffraction Data (D2B, ILL)^a^

Atom	Site	*x*	*y*	*z*	*B* (Å^2^)	Occ.
(a) SXRD (ID22, ESRF):*a* = 5.3810(3) Å, *b* = 5.4543(4) Å, *c* = 20.417(1) Å, *V* = 599.22(7) Å^3^; *R*_Bragg_ = 5.75%						
La1/Pr1 fraction	4*c* (inner RS)	0.25000	0.25185(37)	0.50000	0.637(15)	0.722/0.278
La2/Pr2 fraction	8*f* (outer RS)	0.25000	0.25860(17)	0.31887(1)	0.648(12)	0.639/0.361
Ni	8*f*	0.25000	0.25237(51)	0.09720(4)	0.461(16)	1.000
O1	4*c* (apical inner)	0.25000	0.3165(16)	0.00000	1.644(12)	1.000
O2	8*f* (apical outer)	0.25000	0.2249(17)	0.20950(22)	1.114(11)	1.000
O3	8*e* (equatorial)	0.00000	0.50000	0.10731(32)	1.43(16)	1.000
O4	8*d* (equatorial)	0.50000	0.00000	0.08785(34)	1.58(17)	1.000
(b) NPD (D2B, ILL): *a* = 5.3769(1) Å,*b* = 5.4548(2) Å,*c* = 20.3990(8) Å,*V* = 598.3(1) Å^3^;*R*_Bragg_= 4.11%						
La1/Pr1 fraction	4*c* (inner RS)	0.25000	0.256(3)	0.50000	1.30(16)	0.722/0.278(19)
La2/Pr2 fraction	8*f* (outer RS)	0.25000	0.253(1)	0.3166(18)	0.70(10)	0.639/0.361(19)
Ni	8*f*	0.25000	0.250(1)	0.09575(15)	0.96(8)	1.000
O1	4*c* (apical inner)	0.25000	0.304(2)	0.00000	1.48(18)	1.000
O2	8*f* (apical outer)	0.25000	0.214(1)	0.2035(4)	1.68(11)	1.000
O3	8*e* (equatorial)	0.00000	0.50000	0.1056(3)	0.99(12)	1.000
O4	8*d* (equatorial)	0.50000	0.00000	0.0846(3)	0.89(13)	1.000

a Occupancies are given as site-normalized fractions (unity for a fully occupied site); for the mixed La/Pr positions, the two fractions add up to 1.000 at each site. The occupancies of the four independent oxygen sites refined from the NPD data are stoichiometric within the standard deviations; the resulting composition is La_2_PrNi_2_O_7.00_.

The two-phase SXRD Rietveld refinement converged to *R*_Bragg_ = 5.75% (main phase) and *R*_Bragg_ = 9.12% (La_2_NiO_4_ impurity), with *R*_wp_ = 10.2% and χ^2^ = 368, indicating good agreement between the observed and calculated profiles. Figure 1 illustrates the Rietveld plot from the SXRD data collected at ID22.

**Figure 1:**
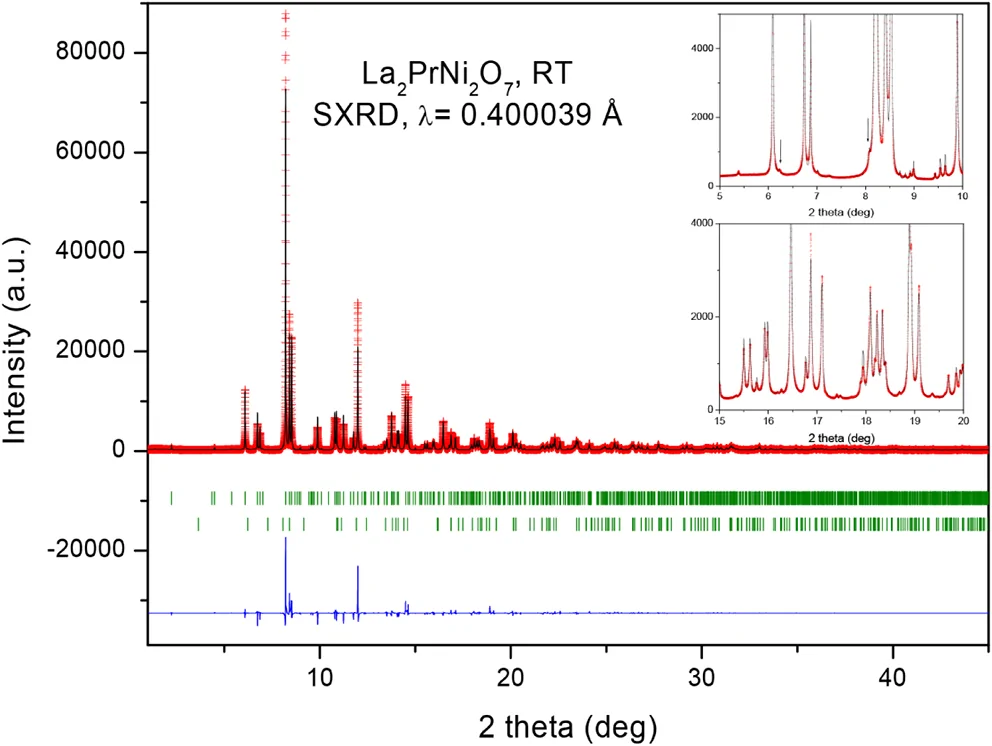
SXRD profiles for La_2_PrNi_2_O_7_ collected on the high-resolution ID22 diffractometer at ESRF at RT. The red crosses correspond to the experimental points; the calculated profile is in black and the difference in blue at the bottom. The green vertical ticks indicate the Bragg positions of La_2_PrNi_2_O_7_ (*Amam*, upper row) and of the La_2_NiO_4_ minor impurity (*Fmmm*, lower row). The two insets illustrate the goodness of the fit in the low- and medium-angle regions; the arrows in the enlarged 6–10° 2θ inset mark the (111) and (113) reflections of La_2_NiO_4_.

It should be noted that the centrosymmetric *Amam* description of bilayer nickelates has recently been questioned. A single-crystal synchrotron study of La_3_Ni_2_O_7_ has revealed very weak reflections that violate the *Amam* extinction conditions and has proposed a polar *Am2m* structure (a setting of space group No. 38) with two inequivalent Ni sites and a checkerboard charge order;^28^ related symmetry lowering has been discussed for other Ruddlesden–Popper nickelates.^29^ The reflections that discriminate between the two models are reported to be nearly 4 orders of magnitude weaker than the main Bragg peaks and are therefore below the detection limit of the present powder diffraction data. The *Amam* model used here should thus be regarded as a description of the average structure, which is fully adequate for the lattice metrics, the oxygen content, and the Ni–O–Ni geometry discussed in this work, but which cannot exclude a subtle *Am2m* distortion at the local or single-crystal level.

### 3.2 Oxygen Stoichiometry from High-Resolution NPD

The high sensitivity of NPD to the oxygen sublattice allowed us to investigate the possible presence of nonstoichiometric oxygen in the interstitial region. The ambient-pressure D2B refinement could not find evidence of any residual nuclear density within the unit cell via difference Fourier synthesis. Figure 2 displays the goodness of fit from NPD data at RT; Figure 3 depicts a schematic view of the crystal structure. It consists of bilayer NiO_6_ octahedral slabs separated by double rock-salt (La/Pr)­O layers. The figure highlights the NiO_6_ octahedral tilting at RT.

**Figure 2:**
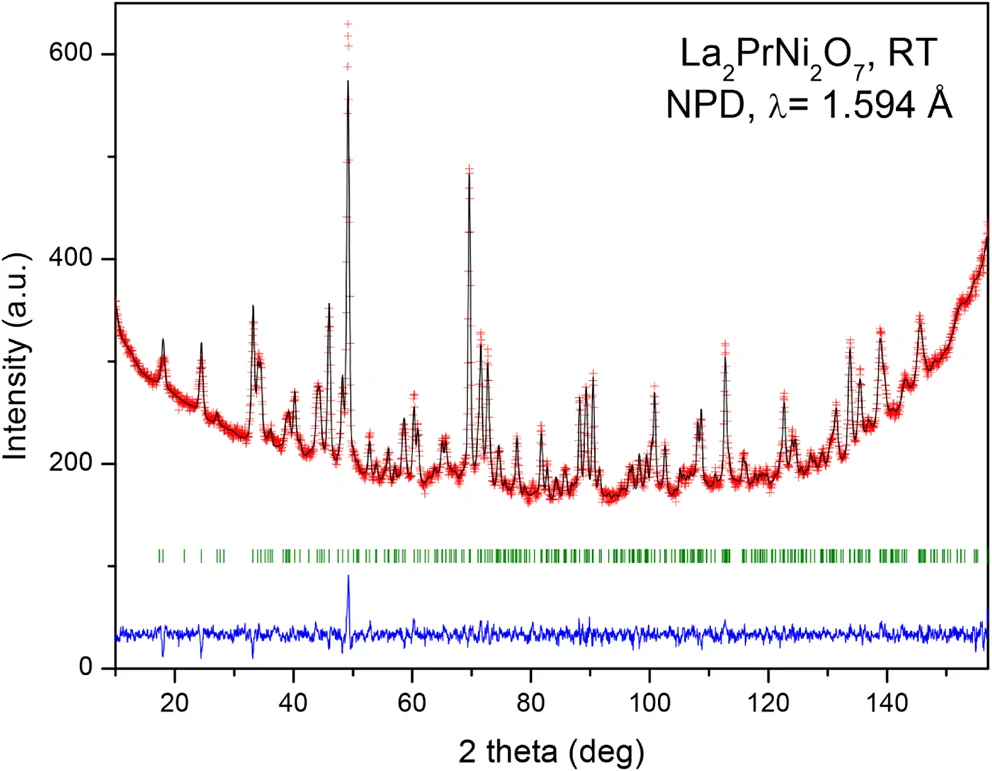
NPD profiles for La_2_PrNi_2_O_7_ collected using the high-resolution D2B diffractometer at RT. The red crosses correspond to the experimental points; the calculated profile is in black and the difference in blue at the bottom; the green ticks indicate the Bragg positions. The region 158.5–160.5° 2θ, containing a weak vanadium container reflection, was excluded from the refinement.

**Figure 3:**
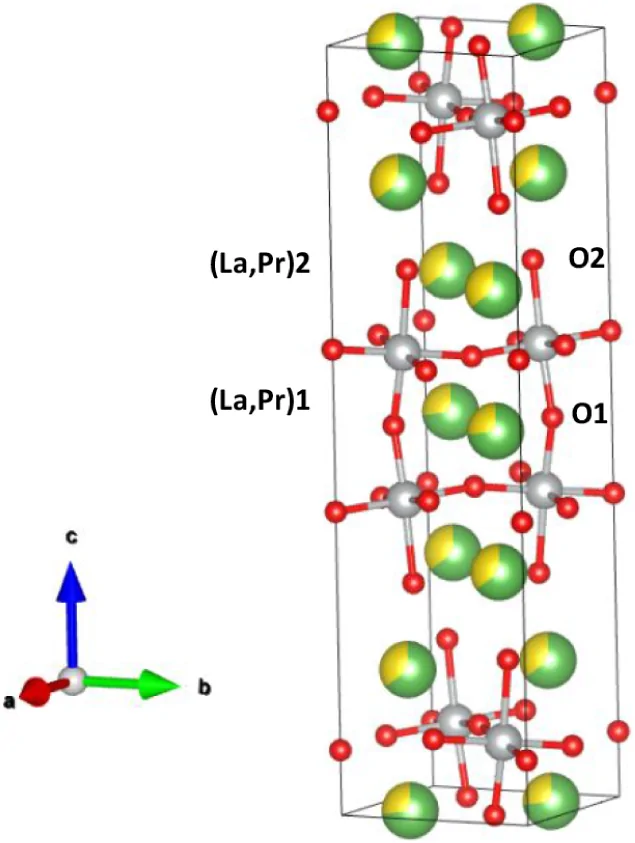
View of the crystal structure of La_2_PrNi_2_O_7+δ_, defined in the *Amam* space group, highlighting the tilting of NiO_6_ octahedra, and the axial O1 atoms linking the octahedra of the bilayer. (La,Pr)­1 and (La,Pr)­2 correspond to the inner (4*c*) and outer (8*f*) positions, respectively. La and Pr are the green and yellow fractions of the (La,Pr) atoms.

The refined occupancy of the O1 site is 1.00(2), and no residual nuclear density was detected at the interstitial 8*g* position by difference Fourier synthesis; the refined occupancy of a test atom placed there was 0.00(2). Summing over the four crystallographically independent oxygen positions gives 7.00(4) O atoms per formula unit, i.e., full stoichiometry within the standard deviations. We note that Wang^7^ reported bulk superconductivity for La_2_PrNi_2_O_7_ samples prepared by the same route and established by TGA and STEM that those samples were free of interstitial oxygen and of intergrowths with other Ruddlesden–Popper phases; the present structural result is therefore compatible with that scenario, although no superconducting property has been measured in the present work and no causal relationship is established here. Moreover, in samples where the oxygen vacancies at the inner apical sites (O1) are healed by mild post-annealing (without introducing interstitial O), the bulk SC fraction approaches ∼97%.^7,9^ The elimination of O1 vacancies and the avoidance of interstitial O uptake are thus the two key oxygen-stoichiometry requirements for robust superconductivity in La_2_PrNi_2_O_7_.^9,11,12^

From the NPD refinement, we can also characterize the NiO_6_ octahedral geometry at ambient conditions. The out-of-plane (apical) Ni–O1 bond distance is 1.975(1) Å, and the in-plane Ni–O3 and Ni–O4 distances are 1.926(6) Å and 1.928(6) Å, respectively. The out-of-plane Ni–O1–Ni bond angle, which is the structural parameter most directly coupled to the d*_z_*_2_ band bandwidth, is 162.8(3)° at ambient pressure, consistent with the orthorhombic tilting of the NiO_6_ polyhedra. These values compare well with those reported from X-ray-based refinements.^5,6,19^

A second critical finding of the NPD refinement is the distribution of La and Pr between the two independent crystallographic sites, given the higher contrast between La and Pr scattering lengths (*b*(La) = 8.24 fm vs. *b*(Pr) = 4.58 fm) than those shown by X-rays (La (*Z* = 57) and Pr (*Z* = 59)). In the *Amam* structure, the 4*c* site (La1/Pr1) corresponds to the inner rock-salt LaO layer adjacent to the NiO_2_ planes, while the 8*f* site (La2/Pr2) is the outer rock-salt layer adjacent to the interstitial region. The Rietveld refinement from NPD data converges to La1:Pr1 normalized occupancies of 0.722(19):0.278(19) at the 4*c* site and to La2:Pr2 = 0.639(19):0.361(19) at the 8*f* site. Both refinements point in the same direction, namely a modest enrichment of Pr at the outer 8f site with respect to the random distribution (Pr fraction of 1/3 at every site). We stress, however, that the departure from the statistical value lies within three standard deviations: for the 4*c* site, the 3σ interval of the La fraction is 0.665–0.779, which encompasses the random value of 2/3. The present data therefore indicate a trend consistent with the EELS observations of Dong,^9^ who reported that Pr dopants preferentially substitute La at the outer rock-salt sites adjacent to the interstitial layer, but they do not allow a statistically firm conclusion on site selectivity; a definitive determination would require either anomalous scattering at the Pr L-III edge or single-crystal data. The driving force proposed by Dong et al., the increased interstitial volume produced by the smaller Pr^3+^ cation (ionic radius *r*(Pr^3+^) = 1.126 Å vs *r*(La^3+^) = 1.160 Å, CN 9),^30,31^ would indeed favor the outer site for Pr, in the same direction as the trend seen here, though not resolved at a statistically significant level by the present data.

### 3.3 Structural Evolution under High Pressure

All HP-NPD patterns in the range 0.08–8.42 GPa refine well in the orthorhombic *Amam* space group (equivalently, *Cmcm* in the setting used during the refinement). No peak splitting, additional reflections, or broadening indicative of a structural phase transition are detected in this pressure range. This is consistent with the SXRD data of Wang^7^ and the DFT calculations of Dong,^9^ which place the *Amam*-to-*I4/mmm* transition at *P*_c_ ≈ 11 GPa for La_2_PrNi_2_O_7_. The present experiment thus maps the structural precursors to the superconducting transition. Figure 4 illustrates the NPD profiles corresponding to different temperatures and pressures, as explained in the caption.

**Figure 4:**
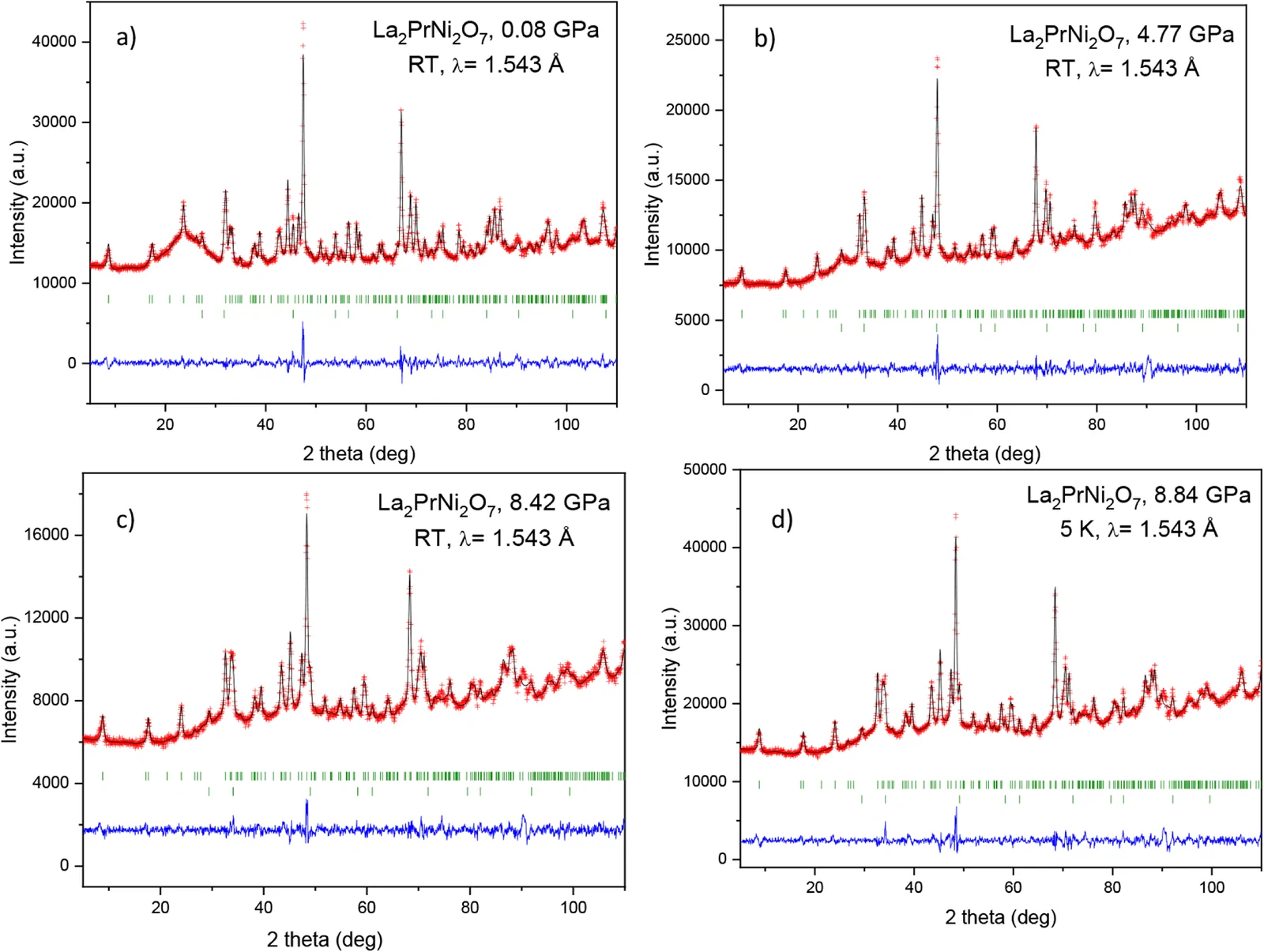
Selected NPD patterns of La_2_PrNi_2_O_7_ collected in a Paris-Edinburgh press under a) 0.08 GPa, RT; b) 4.77 GPa, RT; c) 8.42 GPa, RT; d) 8.84 GPa, 5 K. The red crosses correspond to the experimental points; the calculated profile is in black and the difference in blue at the bottom; the green ticks indicate the Bragg positions; upper row: La_2_PrNi_2_O_7_ (*Amam*), lower row = NaCl (*Fm*3̅*m*) pressure gauge. The broad humps in the low-angle regions arise from incoherent scattering by the 4:1 methanol–ethanol pressure-transmitting medium.

Figure 6 shows the pressure dependence of the normalized unit-cell parameters and volume, together with the orthorhombicity index η. The unit-cell volume decreases monotonically from *V* = 599.9(1) Å^3^ at 0.08 GPa to *V* = 570.1(1) Å^3^ at 8.42 GPa, a reduction of ∼4.97% over this pressure range at RT. The compression is markedly anisotropic. Over the range 0.08–8.42 GPa, the fractional contractions are Δ*a*/*a* = −1.37%, Δ*b*/*b* = −2.07%, and Δ*c*/*c* = −1.61%, so that the softest direction is the in-plane *b*-axis and the stiffest is the in-plane *a*-axis, with the stacking direction *c* intermediate. The preferential contraction of the *b*-axis with respect to *a* is the direct metric signature of the progressive suppression of the orthorhombic distortion, as shown by the *b*/*a* ratio, which decreases from 1.0145 to 1.0073 as the structure approaches the tetragonal phase. Wang^7^ reported an orthorhombic–tetragonal transition at *P*_c_ ≈ 11 GPa for the same composition, consistent with the trend observed here.

The orthorhombicity index η = 2­(*b – a*)/(*b* + *a*) × 100 decreases from 1.43% at 0.08 GPa to 0.73% at 8.42 GPa (Figure 6d), confirming a systematic approach toward tetragonal symmetry with pressure. A linear extrapolation of η­(*P*) reaches zero at approximately 14–15 GPa, slightly above *P*_c_ ≈ 11 GPa. This is physically consistent, since the *Amam*-to-*I*4/*mmm* transition is first-order, and the lattice parameters *a* and *b* may change discontinuously at the transition, causing η to abruptly drop to zero. This index is correlated with the amplitude of the NiO_6_ octahedral tilt, which is the primary order parameter of the *Amam*-to-*I4/mmm* displacive transition. The straightening of octahedral tilts under pressure is a prerequisite for the emergence of the high-symmetry SC phase. Figure 5 illustrates the pressure evolution of the Ni–O1–Ni angle in the intralayer space.

**Figure 5:**
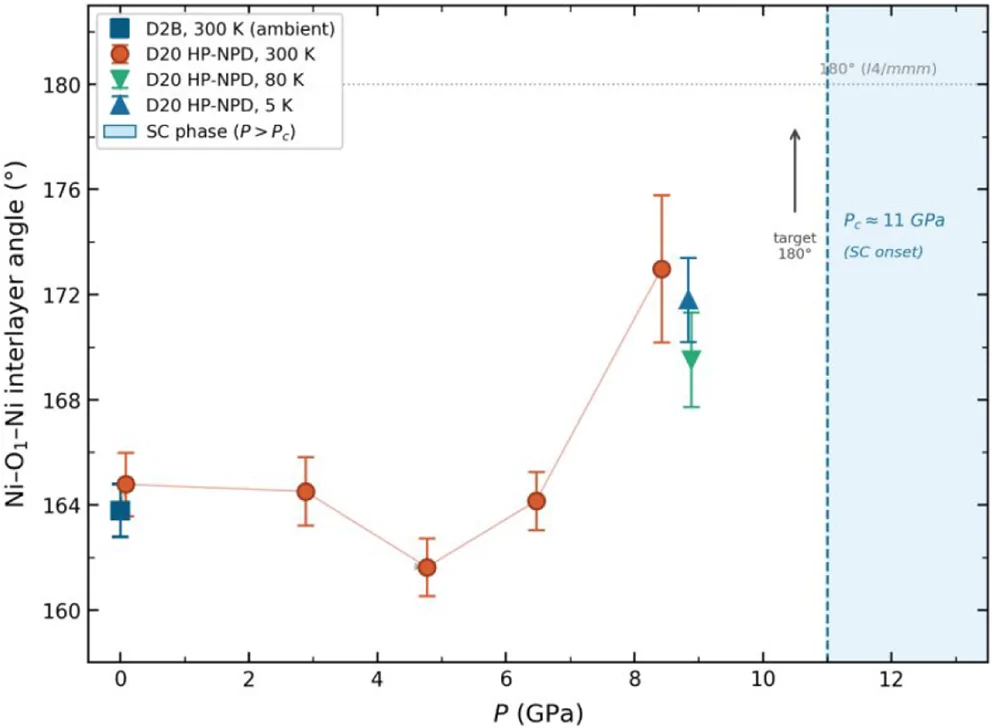
Pressure variation of the tilting Ni–O1–Ni angle within the double layers of NiO_6_ octahedra, closely related to the occurrence of superconductivity. The angle stays nearly constant at ∼164° from 0 to 6.5 GPa; between 6.5 and 8.4 GPa, there is a sharp increase to ∼173°, reflecting the progressive unbuckling of the NiO_6_ octahedra under compression. The low-temperature points (80 and 5 K) at 8.84/8.88 GPa follow the same trend as the 300 K value. Extrapolation toward 180° is consistent with the *Amam* → *I4*/*mmm* structural transition at *P*_c_ ≈ 11 GPa (shaded region), which is the prerequisite for the superconducting phase.

The Ni atom fractional coordinate along *c* (*z*Ni; in the *Cmcm* setting, *x*Ni) evolves from 0.0944(3) at 0.08 GPa to 0.0970(3) at 8.42 GPa, which in the *Amam* convention corresponds to a progressive displacement of Ni away from the midplane of the bilayer. The axial O1 atom shifts toward *y* = 0.275 from *y* = 0.280, consistent with a gradual unbuckling of the equatorial NiO_4_ plane. These changes are entirely continuous, and no structural discontinuity is observed, again in agreement with the second-order (or weakly first-order) character of the *Amam*–*I4*/*mmm* transition predicted by group-theoretical analysis.

The bulk modulus *B*_0_ can be estimated from the *V*(*P*) data by fitting a third-order Birch–Murnaghan equation of state, according to

$$P=\frac{3B_{0}}{2}\left[{\left(\frac{V_{0}}{V}\right)}^{7/3}-{\left(\frac{V_{0}}{V}\right)}^{5/3}\right]\times \left\{1+\frac{3}{4}\left(B^{\prime }-4\right)\left[{\left(\frac{V_{0}}{V}\right)}^{2/3}-1\right]\right\}$$

Fitting the pressure-dependent volumes measured on D20 with *V*_0_, *B*_0_, and *B*_0_′ as free parameters, we obtain *B*_0_ = 135(4) GPa, together with *V*_0_ = 600.5(8) Å^3^ and *B*_0_′ = 7.0(1.2). The quoted uncertainties were obtained from the variance–covariance matrix of the nonlinear least-squares fit and verified by a bootstrap resampling procedure (1000 resamples within the experimental esds). It should be noted that *B*_0_ and *B*_0_′ are strongly correlated when the fitted pressure range (0–8.42 GPa) is small compared with *B*_0_; the elevated value of *B*_0_′ should, therefore, be regarded as an effective parameter of the fit rather than as a physically determined quantity. These values lie within the range of those reported for La_3_Ni_2_O_7_ (125–145 GPa) from synchrotron measurements.^5,6,19^ The moderate compressibility of the bilayer RP structure reflects the stiff NiO_6_ octahedral framework and is consistent with the high pressures required to induce the structural phase transition..

**Figure 6:**
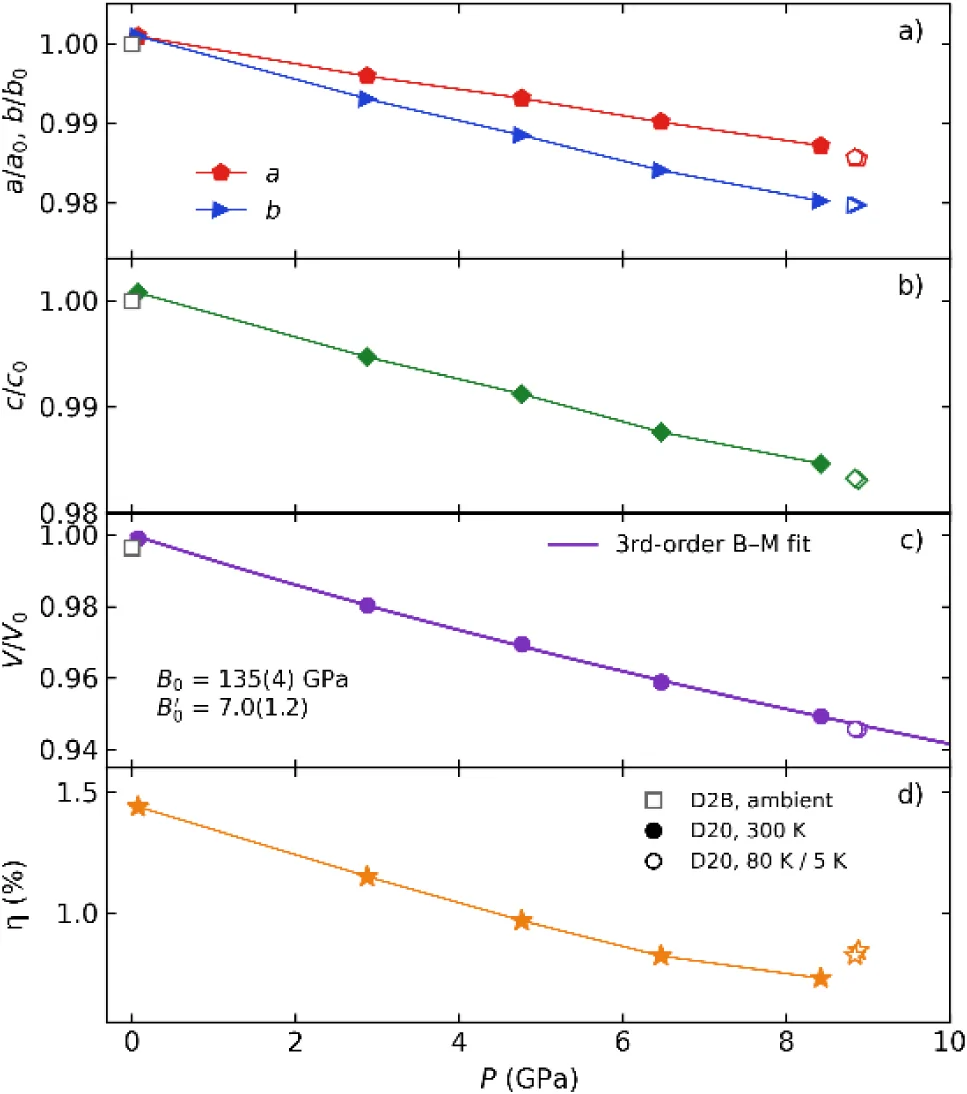
Pressure dependence of (a) the normalized in-plane parameters *a*/*a*_0_ and *b*/*b*_0_, (b) *c*/*c*_0_, (c) *V*/*V*_0_, together with the third-order Birch–Murnaghan fit (solid line; *V*_0_ = 600.5(8) Å^3^, *B*_0_ = 135(4) GPa, *B*_0_′ = 7.0(1.2)), and (d) the orthorhombicity index η. *a*_0_, *b*_0_, and *c*_0_ are the ambient-pressure D2B values; *V*_0_ is the zero-pressure volume of the fit. Full symbols: D20 data at 300 K; open symbols: D20 data at 80 and 5 K; open squares: D2B data at ambient pressure.

### 3.4 Effect of Temperature at High Pressure

After the room-temperature pressure run, the PE press was maintained at 1500 bar, and the sample was cooled first to 80 K and then to 5 K; the internal pressure probed by NaCl shifted to 8.84 GPa. The refined lattice parameters show only marginal changes: the unit-cell volume decreases from 570.1(1) Å^3^ at 300 K to 567.9(1) Å^3^ at 80 K and to 568.0(1) Å^3^ at 5 K (Table 2). The absence of any abrupt volume anomaly or symmetry change down to 5 K indicates that the present sample does not enter the superconducting phase under these conditions, consistent with *P* < *P*_c_ ≈ 11 GPa. The very small thermal contraction between 300 and 5 K (Δ*V*/*V* ≈ 0.36%) is also consistent with the rigid character of the NiO_6_ octahedral framework under pressure.

**Table 2:** Lattice Parameters and Rietveld Agreement Factors for La_2_PrNi_2_O_7_ from SXRD (ID22), D2B-NPD, and D20 HP-NPD^a^

*P* (GPa)	*T* (K)	*a* (Å)	*b* (Å)	*c* (Å)	*V* (Å^3^)	*R*_Bragg_ (%)	χ^2^/*R*_wp_ (%)
Ambient (D2B)	300	5.37691(16)	5.45478(17)	20.3990(8)	598.3(1)	4.11	1.47/13.9
Ambient (SXRD)	300	5.38102(3)	5.45426(4)	20.41668(14)	599.2(1)	5.87	3.92/15.6
0.08	300	5.38332(7)	5.46084(8)	20.4189(3)	600.2(1)	4.95	8.97/16.9
2.88	300	5.35463(9)	5.41493(10)	20.2933(3)	588.4(1)	6.42	4.42/18.5
4.77	300	5.33720(10)	5.39256(11)	20.2219(4)	582.0(1)	5.89	4.35/19.5
6.47	300	5.32528(10)	5.37040(10)	20.1446(4)	576.1(1)	4.48	3.50/19.2
8.42	300	5.31016(11)	5.34898(12)	20.0908(4)	570.7(1)	5.79	3.84/19.4
8.88	80	5.299(1)	5.344(1)	20.054(3)	567.9(1)	6.28	8.13/18.4
8.84	5	5.300(1)	5.344(1)	20.057(4)	568.0(1)	6.95	5.10/20.2

a All HP parameters were converted to *Amam* setting. NaCl was used as the pressure gauge in HP experiments.

Notably, the atomic displacement parameters (*B*_iso_) of all atoms remain positive and physically reasonable under all P–T conditions. The B values of the apical O1 and equatorial O2 oxygen atoms are systematically larger than those of the metal positions, consistent with their higher vibrational freedom in the framework structure. At 5 K and 8.84 GPa, the *B*_iso_ values decrease relative to the room-temperature values, as expected from the quenching of thermal motion. These observations confirm the internal consistency of the refinements and the absence of spurious residues that might indicate a hidden structural anomaly above this pressure.

It is noteworthy that, despite the absence of a bulk structural phase transition in this P range, subtle changes in the Ni–O–Ni bond angle and in the tilt amplitude of the NiO_6_ octahedra occur continuously from 0.08 to 8.42 GPa. These structural precursors are directly relevant to the theoretical models of superconductivity in bilayer nickelates. The tight-binding model of Luo^23^ and the DFT+U calculations of Dong^9^ both predict that the γ-band crossing at the Fermi level—a prerequisite for the d*_z_*_2_-mediated superconducting pairing—requires the Ni–O–Ni bond angle to approach 180°, which occurs only when the octahedral tilt is fully suppressed in the *I4*/*mmm* phase. The present results confirm that, below *P*_c_ ≈ 11 GPa, the bond angle is progressing toward that critical value but has not yet reached it, consistently explaining the absence of superconductivity in the conditions accessed here.

On the other hand, no magnetic scattering above the background noise level was observed in either the D2B (ambient, 300 K) or D20 (high-pressure, 300 K/80 K/5 K) patterns. The absence of magnetic reflections at 300 K is consistent with the paramagnetic state of La_2_PrNi_2_O_7_ under ambient conditions. At 5 K and 8.42 GPa, no additional reflections attributable to magnetic ordering were detected.

## 4 Conclusions

A comprehensive structural study of La_2_PrNi_2_O_7_ has been carried out combining high-resolution synchrotron X-ray (ID22, ESRF) and neutron powder diffraction (D2B and D20, ILL) data from ambient pressure up to 8.42 GPa and down to 5 K, with Rietveld analysis in the orthorhombic *Amam* structure. Regarding the cation distribution, the refined La/Pr occupancies suggest a modest enrichment of Pr at the outer rock-salt La2 (8*f*) site, in the same direction as the EELS results of Dong,^9^ although the effect does not exceed three standard deviations and is therefore not statistically conclusive. Pr^3+^ is not demonstrably ordered over the La1 (4*c*) and La2 (8*f*) sites at the present level of precision. High-resolution NPD identifies full oxygen stoichiometry within the standard deviations, with no interstitial oxygen detected, which is compatible with previous studies,^7^ having shown the near absence of intergrowth with other R-P phases and the lack of extra interstitial oxygen by TGA and STEM measurements. These results are compatible with the sample conditions reported in ref 7 to yield bulk superconductivity—near-stoichiometric oxygen content and minimal Ruddlesden–Popper intergrowth—while the causal link between these structural features and superconductivity itself lies beyond the scope of the present diffraction study. Regarding the high-pressure structural evolution, the orthorhombic *Amam* structure is retained up to 8.42 GPa, below the transition pressure *P*_c_ ≈ 11 GPa. Compression is anisotropic and nearly monotonic: the unit-cell volume decreases by ∼4.97% and the orthorhombicity index η reduces from 1.43% to 0.73%, indicating a continuous structural approach toward the tetragonal phase. The estimated bulk modulus *B*_0_ = 135(4) GPa, with *B*_0_′ = 7.0(1.2), lies within the range of values previously reported for La_3_Ni_2_O_7_. Finally, upon cooling from 300 to 5 K at constant pressure produces only a marginal volume contraction (Δ*V*/*V* ≈ 0.36%) with no symmetry change or anomalous structural behavior, consistent with the absence of the superconducting phase below *P*_c_. These results provide precise structural benchmarks for theoretical models of high-*T*_c_ superconductivity in bilayer nickelates and underline the dual importance of controlling both inner apical oxygen vacancies and interstitial oxygen doping for the realization of robust bulk superconductivity in La_2_PrNi_2_O_7_.

## Data Availability

Embargo periods may apply. Neutron data described in this article are available at the Institut Laue-Langevin (ILL) repository at 10.5291/ILL-DATA.5-24-768—ILL Data Citation; 10.5291/ILL-DATA.5-24-760—ILL Data Citation.
